# Inferior outcome of allogeneic stem cell transplantation for secondary acute myeloid leukemia in first complete remission as compared to de novo acute myeloid leukemia

**DOI:** 10.1038/s41408-020-0296-3

**Published:** 2020-03-03

**Authors:** Ann-Kristin Schmaelter, Myriam Labopin, Gerard Socié, Maija Itälä-Remes, Didier Blaise, Ibrahim Yakoub-Agha, Edouard Forcade, Jan Cornelissen, Arnold Ganser, Dietrich Beelen, Hélène Labussière-Wallet, Jakob Passweg, Bipin N. Savani, Christoph Schmid, Arnon Nagler, Mohamad Mohty

**Affiliations:** 1 Department of Hematology and Oncology, Augsburg University Hospital, Augsburg, Germany; 2 Department of Haematology, Saint Antoine Hospital, Université Pierre et Marie Curie, INSERM UMR 938 Paris, France; 3 EBMT Paris study office/ CEREST-TC, Paris, France; 4 Hopital St. Louis, Dept.of Hematology – BMT, Paris, France; 5 HUCH Comprehensive Cancer Center, Stem Cell Transplantation Unit, Helsinki, Finland; 6 Programme de Transplantation & Therapie Cellulaire, Centre de Recherche en Cancérologie de Marseille, Institut Paoli Calmettes, Marseille, France; 7 CHU de Lille, LIRIC, INSERM U995, Université de Lille, 59000 Lille, France; 8 CHU Bordeaux Hôpital Haut-Leveque, Pessac, France; 9 Erasmus MC Cancer Institute, University Medical Center Rotterdam, Department of Hematology, Rotterdam, The Netherlands; 10 Hannover Medical School, Department of Haematology, Hemostasis, Oncology and Stem Cell Transplantation, Hannover, Germany; 11 University Hospital, Department of Bone Marrow Transplantation, Essen, Germany; 12 Centre Hospitalier Lyon Sud, Service Hematologie, Lyon, France; 13 University Hospital, Hematology, Basel, Switzerland; 14 Division of Hematology/Oncology, Vanderbilt University Medical Center, Nashville, TN USA; 15 Hematology Division, Chaim Sheba Medical Center, Tel Hashomer, Israel

**Keywords:** Stem-cell research, Cancer stem cells

## Abstract

Following chemotherapy, secondary acute myeloid leukemia (sAML), occurring after antecedent hematologic diseases, previous chemotherapy or radiation, has an inferior prognosis compared with de novo AML. To define the outcome of sAML in the context of allogeneic stem cell transplantation (alloSCT), a retrospective, registry-based comparison was performed, including 11,439 patients with de novo and 1325 with sAML. Among transplants in first complete remission (CR1) (*n* = 8,600), the 3-year cumulative incidence of relapse (RI) and non-relapse mortality (NRM) was 28.5% and 16.4% for de novo, and 35% and 23.4% for sAML. Three-year overall survival (OS), leukemia-free survival (LFS) and Graft-versus-Host Disease/relapse-free survival (GRFS) was 60.8%, 55.1%, and 38.6% for de novo, and 46.7%, 41.6%, and 28.4% for sAML, respectively. In multivariate analysis, sAML was associated with a lower OS (HR = 1.33 [95% CI = 1.21–1.48]; *p* < 10^−5^), LFS (HR = 1.32 [95% CI = 1.19–1.45]; *p* < 10^−5^) and GRFS (HR = 1.2 [95% CI = 1.1–1.31]; *p* < 10^−4^) and higher NRM (HR = 1.37 [95% CI = 1.17–1.59]; *p* < 10^−4^) and RI (HR = 1.27 [95% CI = 1.12–1.44]; *p* < 10^−3^). Results of the Cox model were confirmed in a matched-pair analysis. In contrast, results did not differ between de novo and sAML after alloSCT in induction failure or relapse. Hence, this analysis identified sAML as an independent risk factor for outcome after alloSCT in CR1.

## Introduction

Secondary acute myeloid leukemia (sAML) arises from underlying hematological disorders such as myelodysplastic syndrome (MDS), myeloproliferative neoplasm (MPN), MPN/MDS overlap or bone marrow failure syndromes. Furthermore, therapy related AML (tAML), which can develop after a prior treatment by chemotherapy or radiation, can be assigned to the sAML classification^1^.

Treating sAML with conventional chemotherapy or autologous stem cell transplantation is associated with a poor prognosis^2–4^. Therefore, in comparison to de novo AML, sAML is thought to have an inferior prognosis in general^5–7^. Allogeneic stem cell transplantation (alloSCT) is regarded as the treatment option for sAML with the best chance of achieving a long-term remission. Considering the transplant setting^8,9^, patients with sAML tend to have less available HLA identical siblings, and the majority receive reduced intensity conditioning (RIC)^10,11^. However, it is not clear whether having sAML per se is a risk factor for outcome after alloSCT, when all well-known risk factors are controlled for^8^.

So far, only smaller studies have addressed this question^12^. Therefore, the Acute Leukemia Working Party (ALWP) of the European Society of Blood and Marrow Transplantation (EBMT) performed a retrospective registry-based analysis, comparing the outcome of adult patients diagnosed with sAML and de novo AML, who had received alloSCT.

## Methods

### Study design

Data were provided by the EBMT registry, which comprises more than 600 transplant centers providing reports and annual follow-up on all consecutive stem cell transplantations. Audits are routinely performed to determine the accuracy of the data. Since 1990, patients have provided informed consent, authorizing the use of their personal information for research purposes. The study was approved by the general assembly and review board of the ALWP.

Inclusion criteria were age ≥ 18 years, alloSCT between January 2000 and December 2016 from a matched related, 9/10 or 10/10 antigen matched unrelated, or a T-cell replete haploidentical donor for either de novo or sAML, and available information on cytogenetics. The number of patients fitting inclusion criteria was 11,439 patients with de novo AML and 1325 with sAML.

Variables of interest were patient and donor characteristics (age, gender, Karnofsky performance score [KPS], cytomegalovirus [CMV] serostatus), disease-related (favorable/intermediate/adverse cytogenetics according to the British Medical Research Council classification^13^, remission status at alloSCT) and transplant-related factors (graft source, donor type, conditioning, T-cell depletion [TCD] and graft-versus-host disease [GVHD] prophylaxis). Outcomes comprised overall survival (OS), leukemia free survival (LFS), GVHD/relapse-free survival (GRFS), cumulative incidence of relapse (RI), non-relapse mortality (NRM), acute and chronic GVHD, and cause of death.

### Definitions

Secondary AML was defined as AML with an antecedent MDS, MPN or other malignant hematologic disorder, bone marrow failure syndrome, or solid tumor with prior chemotherapy or radiation^8^. For a subgroup analysis, tAML was defined by previous treatment of solid tumors, Hodgkin or non-Hodgkin Lymphoma (NHL), acute lymphoblastic leukemia (ALL) or chronic lymphocytic leukemia (CLL) using chemotherapy or radiation^14^.

As recommended complete remission (CR) was defined by <5% bone marrow (BM) blasts, absence of circulating blasts and extramedullary disease. Failure to achieve CR after two courses of standard induction chemotherapy was defined as primary induction failure (PIF). Relapse was defined by more than 5% BM blasts or reappearance of circulating blasts after a documented CR^15^. OS was defined as the interval between day of alloSCT and day of death or last follow-up, LFS as interval between alloSCT and date of leukemia persistence, relapse or progression. NRM was defined as death from any cause without relapse or progression. GRFS was defined as absence of acute GVHD III-IV, chronic GVHD requiring systemic treatment, relapse, or death^16,17^. Reduced intensity conditioning (RIC) was defined using EBMT criteria^18^. Cytogenetic subgroups were defined according to SWOG criteria^19^.

### Statistical analysis

Descriptive statistics used median, inter-quartile range (IQR), minimum (min) and maximum (max) for continuous data, counts and percentages for categorical variables. Patient, disease, and transplant-related characteristics for the two cohorts (de novo or secondary AML) were compared by using χ^2^ statistics for categorical variables and the Mann-Whitney test for continuous variables. The date of transplantation was the starting point for time-to-event analysis. Survivors were censored at last contact. Cumulative incidence was used to estimate the endpoints of NRM, RI, acute and chronic GVHD to accommodate for competing risks. Relapse and death were considered competing events for acute and chronic GVHD. Probabilities of OS, LFS, and GRFS were calculated using the Kaplan–Meier method. Univariate analyses were done using the Gray’s test for cumulative incidence functions and the log rank test for OS, GRFS, and LFS.

A multivariate Cox proportional-hazards model was performed to account for imbalances of risk factors between the two cohorts. Due to a significant interaction between disease stage at transplant and diagnosis (de novo or sAML), the analysis was stratified by stage at alloSCT. All variables associated with a significant outcome in univariate analysis (*p* < 0.05), factors known from the literature to have a potential influence on outcome, and variables not equally balanced between cohorts were included in the Cox model.

To confirm the results from the Cox model, a matched-pair analysis of secondary versus de novo AML was performed, using the following criteria for matching: Age (±3 years), cytogenetics, stage at alloSCT, KPS, conditioning, in and ex vivo TCD, donor type, donor/recipient gender and CMV serostatus combinations, and graft source.

Results were expressed as hazard ratio (HR) and 95% confidence intervals (CI). Proportional hazards assumptions were checked for all models using the Grambsch-Therneau residual-based test. All tests were 2-sided. The Type I error rate was fixed at 0.05 for the determination of factors associated with time-to-event outcomes. Statistical analyses were performed with SPSS 24.0 (SPSS Inc, Chicago, IL) and R 3.4.1 (https://www.R-project.org/).

## Results

### Patients’ disease and transplant characteristics

Using the criteria defined above, 11,439 patients with de novo AML and 1325 with sAML were included. Median follow-up after alloSCT was 36.1 and 33.1 months for de novo and sAML, respectively. Patients’ disease- and transplant-related characteristics are outlined in Table 1. Among patients with sAML, 825 (62.5%) had evolved from previous myeloid malignancy (most frequently MDS [68.5%], MPN [11.0%], and MDS/MPN overlap [13.3%]) or bone marrow failure syndrome. In 500 (37.7%) patients sAML was treatment related, following chemotherapy or radiation for other types of cancer, with breast cancer (38.2%) being the most frequent malignancy, followed by lymphoma (33.2%), other solid tumors (26.2%), ALL (2.4%), and CLL and myeloma (<1% each). In the de novo AML and sAML cohorts 6306 (55.1%) and 574 (43.3%), respectively, received a myeloablative conditioning regimen (MAC), whereas 5,133 (44.9%) of de novo AML patients and 751 (56.7%) of sAML patients were treated with a RIC regimen (cf. supplemental Tables 1–2 for details on conditioning and immunosuppression).

**Table 1:** Patient, disease and transplant characteristics in entire population.

Characteristic	de novo AML	sAML	*p*
*N*	11439 (89.62%)	1325 (10.38%)	
Age at SCT (year), median, (range) (IQR)	49.3 (18–76.8) (37.8–58.2)	57.7 (18.3–76) (49.3–63.5)	<10^−3^
Year of SCT, median (range) (IQR)	2011 (2000–2016) (2007–2014)	2012 (2000–2016) (2009–2014)	<10^−3^
Months diagnosis to SCT, median (IQR)	5.6 (4.1–9.7)	4.7 (3.5–6.3)	<10^−3^
Status at SCT			
CR1	7691 (67.23%)	909 (68.6%)	<10^−3^
CR2	2132 (18.64%)	93 (7.02%)	
PIF	607 (5.31%)	199 (15.02%)	
Relapse	1009 (8.82%)	124 (9.36%)	
Donor to patient sex			
No female to male	9188 (80.32%)	1096 (82.72%)	0.037
Female to male	2251 (19.68%)	229 (17.28%)	
KPS at SCT			
KPS < 80	553 (4.83%)	94 (7.09%)	<10^−3^
KPS ≥ 80	10,886 (95.17%)	1231 (92.91%)	
Donor			
MSD	6405 (55.99%)	578 (43.62%)	<10^−3^
UD 10/10	3281 (28.68%)	518 (39.09%)	
UD 9/10	1035 (9.05%)	139 (10.49%)	
Haploidentical donor	718 (6.28%)	90 (6.79%)	
CMV status donor (D) and recipient (R)			
D−/R−	2725 (23.82%)	300 (22.64%)	0.005
D+/R−	1060 (9.27%)	112 (8.45%)	
D−/R+	2524 (22.06%)	350 (26.42%)	
D+/R+	5130 (44.85%)	563 (42.49%)	
Cytogenetics			
Favorable	1492 (13.04%)	58 (4.38%)	<10^−3^
Intermediate	7809 (68.27%)	836 (63.09%)	
Adverse	2138 (18.69%)	431 (32.53%)	
Graft source			
Bone marrow	2005 (17.53%)	162 (12.23%)	<10^−3^
Peripheral blood	9431 (82.47%)	1163 (87.77%)	
Conditioning regimen			
MAC	6306 (55.13%)	574 (43.32%)	<10^−3^
RIC	5133 (44.87%)	751 (56.68%)	
T-cell depletion (TCD)			
No in vivo TCD	5951 (52.02%)	502 (37.89%)	<10^−3^
In vivo TCD	5488 (47.98%)	823 (62.11%)	
No ex vivo TCD	10964 (95.85%)	1305 (98.49%)	<10^−3^
Ex vivo TCD	475 (4.15%)	20 (1.51%)	

Graft source: 3 missing in de novo AML group.

Details on conditioning regimes are presented in supplemental Table S1.

Details on immunosuppression are presented in supplemental Table S2.

*SCT* stem cell transplantation, *IQR* interquartile range, *CR* complete remission, *PIF* primary induction failure, *KPS* Karnofsky performance status, *MSD* matched sibling donor, *UD* unrelated donor, *CMV* cytomegalovirus, *MAC* myeloablative conditioning, *RIC* reduced-intensity conditioning, *TCD* T-cell depletion.

### Outcomes

Engraftment was achieved in 11,133 (98.2%) and 1264 (96.6%) patients with de novo AML and sAML, respectively. Three-year OS and LFS rates for the entire cohort were 54.6% [53.6–55.6] and 48.9% [47.9–49.9] for de novo AML and 43.1% [40.1–46] and 37.9% [35.1–40.8] for sAML. Three-year NRM, RI, and GRFS were 18% [17.3–18.8], 33% [32.1–34], and 34.3% [33.3–35.3] respectively, for de novo and 24.8% [22.4–27.3], 37.3% [34.5–40.1], and 25.8% [23.1–28.4], respectively, for sAML. Overall incidence of acute GvHD grades II–IV and chronic GvHD were 23.5% [22.7–24.3]/40.6% [39.6–41.6] for de novo AML and 24.4% [22–26.8]/35.4% [32.6–38.1] for sAML. The most frequent cause of death was the reoccurrence of the original disease, other causes of death are shown in supplemental Table S3.

### Comparison between de novo and secondary AML

As mentioned above, a significant interaction between disease status at transplant and diagnosis (de novo or sAML) was observed. Therefore, the analysis of the role of sAML had to be stratified according to disease status at time of alloSCT. We focused on the comparison of patients transplanted in CR1, primary refractory phase and relapse, as only 93 patients with sAML were reported to have been transplanted in CR2, precluding relevant adjustment in that subgroup.

### Transplantation in first complete remission

**Fig. 1:**
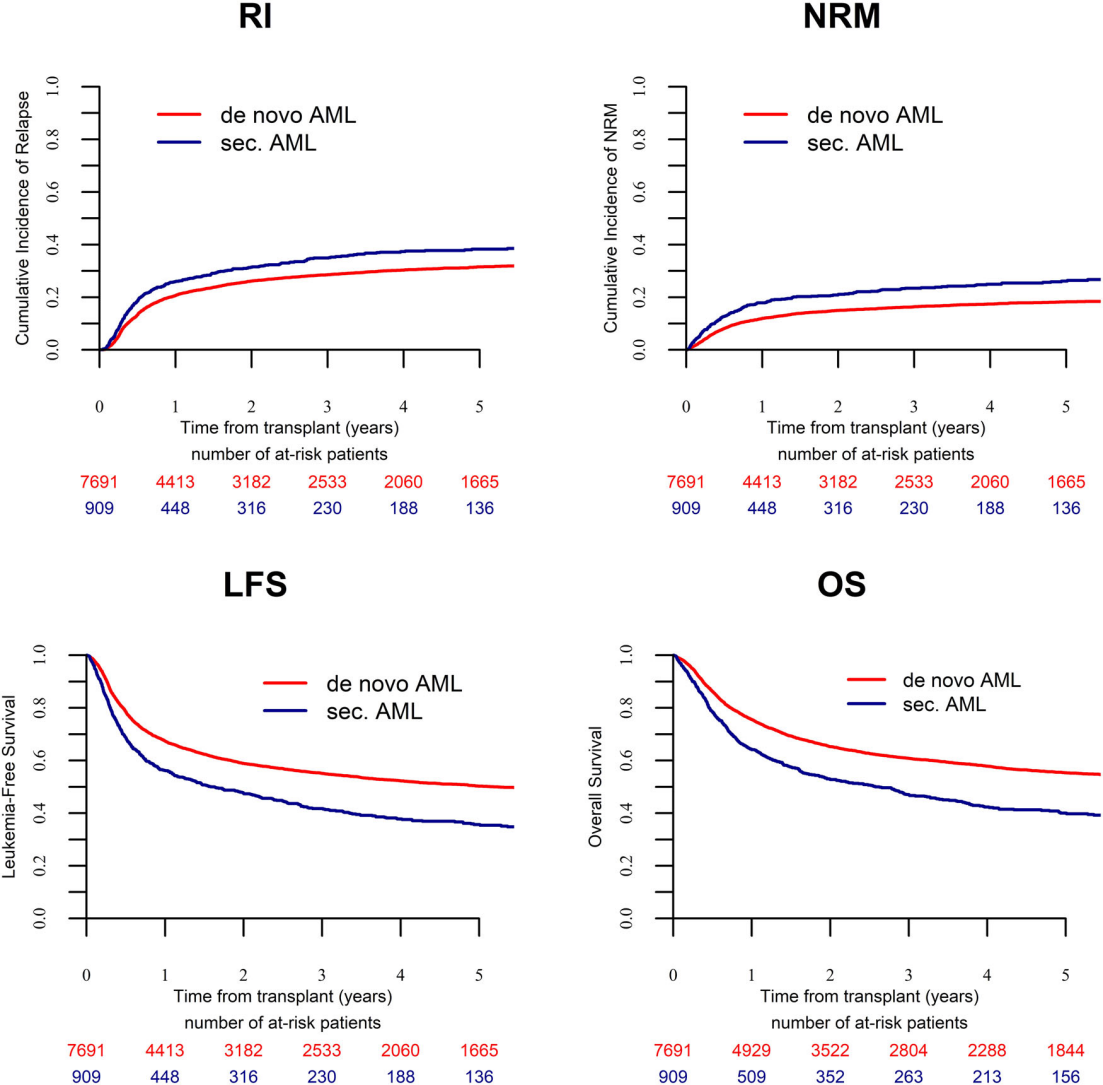
Outcome of patients with either de novo or secondary AML after alloSCT in CR1. RI Incidence of relapse, OS Overall survival, NRM Non-relapse mortality, LFS Leukemia-free survival (*p* = 8.6 × 10^−5^, NRM: *p* < 10^−5^, LFS: *p* < 10^−5^, OS: *p* < 10^−5^).

Transplantation in CR1 had been performed in 7691 (67.2%) patients with de novo and 909 (68.6%) with sAML. Three-year cumulative RI and NRM after alloSCT was 28.5% and 16.4% for de novo AML and 35% and 23.4% for sAML, respectively. Three-year OS, LFS and GRFS were 60.8%, 55.1%, and 38.6%, respectively, for de novo and 46.7%, 41.6%, and 28.4%, respectively, for sAML (all *p*-values < 10^–5^) (Fig. 1, Table 2).

**Table 2:** OS, Relapse, NRM, LFS, and GRFS at 3 years (univariate analysis).

		CR1	PIF	Rel
OS	de novo AML	60.8% [59.6–62]	29.8% [25.6–34]	23.6% [20.7–26.4]
	sAML	46.7% [43.1–50.3]	35.7% [28.5–42.9]	24.8% [16.7–33]
	*p* (log rank)	<10^−5^	0.541	0.721
Relapse	de novo AML	28.5% [27.4–29.6]	53% [48.6–57.3]	58.4% [55.1–61.5]
	sAML	35% [31.7–38.4]	43% [35.6–50.1]	49.5% [40–58.2]
	*p* (log rank)	8.6 x 10^−5^	0.05	0.089
NRM	de novo AML	16.4% [15.5–17.3]	21.2% [17.9–24.7]	23% [20.3–25.7]
	sAML	23.4% [20.5–26.4]	27.9% [21.5–34.6]	30.3% [22.2–38.7]
	*p* (log rank)	<10^−5^	0.027	0.115
LFS	de novo AML	55.1% [53.8–56.3]	25.8% [21.9–29.7]	18.7% [16.1–21.3]
	sAML	41.6% [38–45.1]	29.1% [22.3–36]	20.3% [12.8–27.8]
	*p* (log rank)	<10^−5^	0.753	0.674
GRFS	de novo AML	38.6% [37.4–39.9]	16.8% [13.5–20.2]	13.8% [11.6–16.1]
	sAML	28.4% [25.1–31.6]	16.2% [10.5–21.9]	16.3% [9.5–23.2]
	*p* (log rank)	<10^−5^	0.981	0.448

*OS* overall survival, *NRM* non-relapse mortality, *LFS* leukemia-free survival, *GRFS* Graft-versus-Host Disease/relapse-free survival, *CR* complete remission, *PIF* primary induction failure, *Rel* relapse.

In a multivariate Cox model, sAML was associated with a lower OS (HR = 1.33 [1.21–1.48]; *p* < 10^–5^). Other prognostic factors for OS after alloSCT in CR1 were age (HR = 1.19 [1.15–1.23]; *p* = < 10^−5^), cytogenetics (intermediate, HR = 1.27 [1.09–1.48]; *p* = 0.002, adverse, HR = 2 [1.7–2.35]; *p* = <10^−5^), patient/donor sex combination (female in male, HR = 1.12 [1.03–1.22]; *p* = 0.007), KPS > 80% (HR = 0.66 [0.56–0.79]; *p* < 10^−5^) and donor type (10/10 matched unrelated donor [UD], HR = 1.14 [1.04–1.24]; *p* = 0.007; 9/10 matched UD, HR = 1.36 [1.19–1.56]; *p* < 10^−5^; haploidentical donor, HR = 1.33 [1.11–1.6]; *p* = 0.002) (Table 3).

**Table 3:** Multivariate analysis of risk factors for overall survival, stratified by stage at transplantation.

	SCT in CR1			SCT in PIF			SCT in relapse		
	HR	CI	*p*	HR	CI	*p*	HR	CI	*p*
sAML	1.33	1.21–1.48	**<10**^**−5**^	0.917	0.726–1.157	0.46499	0.946	0.752–1.192	0.640
Age (per 10 years)	1.18	1.15–1.225	**<10**^**−5**^	1.103	1.025–1.188	**0.009**	1.051	0.994–1.112	0.081
Year of SCT	0.996	0.987–1.006	0.441	0.971	0.949–0.994	**0.015**	0.999	0.981–1.018	0.919
Relapse 2 vs relapse 1	n.a.			n.a.			1.232	1.029–1.475	**0.023**
Favorable cytogenetics (ref)	1			1			1		
Intermediate	1.27	1.088–1.482	**0.002**	n.a.	n.a.		n.a.		
Adverse	1.996	1.696–2.349	**<10**^**−5**^	1.653	1.374–1.988	**<10**^**−5**^	1.368	1.163–1.609	**<10**^**−4**^
Female donor to male recipient	1.124	1.033–1.224	**0.007**	1.255	1.013–1.555	**0.037**	1.155	0.972–1.373	0.102
Previous autograft	1.371	1.012–1.857	**0.041**	n.a.			n.a.		
KPS > 80%	0.662	0.555–0.789	**<10**^**−5**^	0.517	0.405–0.66	**<10**^**−5**^	0.56	0.464–0.676	**<10**^**−5**^
MSD (ref)	1			1			1		
UD 10/10	1.135	1.036–1.243	**0.007**	1.043	0.809–1.345	0.746	0.806	0.668–0.973	**0.025**
UD 9/10	1.361	1.189–1.558	**10**^**−5**^	0.98	0.7–1.371	0.904	1.17	0.933–1.468	0.174
Haploidentical donor	1.333	1.11–1.602	**0.002**	1.304	0.905–1.88	0.154	0.912	0.705–1.179	0.482
PB vs BM	1.002	0.907–1.106	0.976	0.93	0.672–1.286	0.660	0.804	0.648–0.998	**0.048**
Patient CMV-positive	1.094	1.011–1.183	**0.025**	1.24	1.006–1.53	**0.044**	1.028	0.874–1.209	0.738
Donor CMV-positive	0.968	0.899–1.042	0.386	1.048	0.861–1.275	0.643	1.101	0.947–1.28	0.209
MAC vs RIC	1.026	0.94–1.12	0.570	1.01	0.823–1.24	0.923	1.062	0.91–1.239	0.448
In vivo T-cell depletion	0.935	0.859–1.018	0.122	0.887	0.703–1.119	0.311	1.108	0.939–1.308	0.223

*SCT* stem cell transplantation, *CR* complete remission, *PIF* primary induction failure, *HR* hazard ratio, *CI* confidence interval, *ref* reference, *KPS* Karnofsky performance status, *MSD* matched sibling donor, *UD* unrelated donor, *PB* peripheral blood, *BM* bone marrow, *CMV* cytomegalovirus, *MAC* myeloablative conditioning, *RIC* reduced-intensity conditioning.

Statistically significant values are marked in bold.

Regarding other outcome parameters, multivariate analysis showed that sAML was associated with a lower LFS (HR = 1.32 [1.19–1.45]; *p* < 10^−5^) and GRFS (HR = 1.2 [1.1–1.31]; *p* < 10^−4^), a higher NRM (HR = 1.37 [1.17–1.59]; *p* < 10^−3^) and RI (HR = 1.27 [1.12–1.44]; *p* < 10^−4^). Detailed results of a Cox model of risk factors for RI, NRM, LFS, and GRFS are shown in supplemental Tables 4–7. The inferior outcome of patients with sAML was also observed in an exploratory subgroup analysis of patients below and above the age of 60 years (data not shown).

### Separate comparison for AML evolving from myeloid malignancies/bone marrow failure syndromes and treatment-related AML

To evaluate a potential difference among patients with sAML evolving from myeloid malignancies or bone marrow failure syndromes versus treatment-related AML, a second Cox model was fitted, including the two subtypes of sAML as covariates. Both sAML after myeloid malignancies/BM failure and tAML were associated with increased OS, LFS, and GRFS following alloSCT in CR1 when compared to de novo AML (supplemental Table S8).

### Transplantation in primary induction failure or active relapse

Among patients with sAML, 199 (15.02%) and 124 (9.36%) had been transplanted in PIF and relapsed disease, respectively; in de novo AML, 607 (5.3%) had PIF, and 1009 (8.82%) had relapsed disease at time of alloSCT. In contrast to the cohort transplanted in CR1, sAML was not identified as a relevant factor for OS (PIF: HR = 0.92; *p* = 0.465; relapse: HR = 0.95; *p* = 0.64; cf. Tables 2 and 3), nor for RI, NRM, LFS, and GRFS. Risk factors that affected OS, RI, LFS, and GRFS in the cohort transplanted for active disease included cytogenetics and KPS > 80% (cf. Table 3 and supplemental Tables 4–7 for details).

### Role of sAML for GVHD

Independently of disease status before transplantation, sAML was not a risk factor for the occurrence of acute or chronic GVHD. Risk factors for acute GVHD grade II–IV across all subgroups were donor type, female donor for male patient and in vivo TCD. In the CR1 cohort, among others, age, cytogenetics, KPS, and conditioning regime were additional factors. Risk of chronic GVHD was mainly determined by graft source and in vivo TCD. In CR1, age, female donor to male recipient and donor type were additional factors (supplemental Tables 9–10 or details).

### Matched-pair analysis

To confirm the results from the Cox model, a matched-pair comparison was performed between de novo and sAML. Using the criteria mentioned above, 877 well-matched pairs were identified (cf. supplemental Table S11). The matched-pair analysis confirmed the findings of the Cox model: Overall, sAML was associated with a lower 3-year cumulative incidence of OS of 46.9% (95% CI, 43.3–50.5) compared to 53.9% (95% CI, 50.1–57.7) in de novo AML (*p* = 0.01). Furthermore, patients with sAML had a lower LFS (*p* = 0.03), GRFS (*p* = 0.04), and a higher NRM (*p* = 0.01). However, when the analysis was stratified by disease status at alloSCT, again differences were only seen among patients transplanted in CR1 (719 pairs, 82%). The 3-year OS in the CR1 sAML and in the de novo AML cohort was 48.1% (95% CI, 44–52.1) and 57.4% (95% CI, 53.2–61.6) (*p* = 0.0004), respectively. In contrast, no significant difference in OS was observed between secondary and de novo AML after alloSCT in PIF (67 pairs, 7.6%, *p* = 0.58) and relapse (44 pairs, 5%, *p* = 0.20). Results of the matched pair analysis are shown in Table 4.

**Table 4:** OS, Relapse, NRM, LFS, and GRFS at 3 years according to matched-pair analysis.

		Entire cohort	CR1	PIF	Rel
OS	De novo AML	53.9% [50.1–57.7]	57.4% [53.2–61.6]	32.4% [20.1–44.7]	23.9% [9.5–38.3]
	sAML	46.9% [43.3–50.5]	48.1% [44–52.1]	36.3% [23.4–49.2]	33.5% [19.2–47.9]
	HR (95% CI) *p* value	1.25 (1.05–1.49) *p* = 0.01	1.43 (1.17–1.74) *p* < 10^−3^	0.86 (0.50–1.48) *p* = 0.58	0.65 (0.34–1.25) *p* = 0.20
Relapse	De novo AML	34.3% [30.9–37.8]	32.4% [28.6–36.3]	49% [35.5–61.1]	53.7% [37.3–67.6]
	sAML	36.9% [33.5–40.3]	35.9% [32.1–39.7]	45.8% [32.5–58.1]	46.8% [31–61.1]
	HR (95% CI) *p* value	1.08 (0.88–1.33) *p* = 0.44	1.24 (0.98–1.56) *p* = 0.07	0.48 (0.24–0.96) *p* = 0.04	0.87 (0.41–1.82) *p* = 0.71
NRM	De novo AML	16.6% [14–19.4]	15.7% [12.9–18.8]	20.6% [11.2–32]	23.6% [10.8–39]
	sAML	21.3% [18.5–24.2]	21.2% [18.1–24.4]	24.4% [14.2–36.1]	21.4% [10.4–35]
	HR (95% CI) *p* value	1.42 (1.08–1.87) *p* = 0.01	1.47 (1.08–2.0) *p* = 0.01	1.57 (0.61–4.05) *p* = 0.35	0.83 (0.25–2.73) *p* = 0.76
LFS	De novo AML	49.1% [45.4–52.8]	51.8% [47.7–56]	30.4% [18.4–42.5]	22.7% [8.8–36.7]
	sAML	41.8% [38.3–45.4]	42.9% [39–46.9]	29.8% [17.7–41.9]	31.8% [17.6–46]
	HR (95% CI) *p* value	1.19 (1.01–1.40) *p* = 0.03	1.32 (1.10–1.59) *p* = 0.003	0.72 (0.42–1.23) *p* = 0.23	0.86 (0.46–1.61) *p* = 0.63
GRFS	De novo AML	34.3% [30.7–38]	37% [32.9–41]	18.9% [8.4–29.4]	7.5% [0–19.1]
	sAML	28.6% [25.3–32]	29.2% [25.4–32.9]	18.6% [7.8–29.4]	24.8% [11.6–37.9]
	HR (95% CI) *p* value	1.13 (0.97–1.32) *p* = 0.12	1.21 (1.02–1.44) *p* = 0.03	0.82 (0.49–1.36) *p* = 0.44	0.86 (0.47–1.60) *p* = 0.64

*OS* overall survival, *NRM* non-relapse mortality, *LFS* leukemia-free survival, *GRFS* Graft-versus-Host Disease/relapse-free survival, *HR* hazard ration, *CI* confidence interval, *CR* complete remission, *PIF* primary induction failure, *Rel* relapse.

## Discussion

In this large-scale, registry-based analysis, a multivariate model identified sAML as an independent risk factor for OS, LFS, GRFS, RI, and NRM after alloSCT in CR1, as compared to de novo disease. A matched-pair analysis confirmed these findings, which were observed both in treatment-related AML and AML evolving from other myeloid disorders or BM failure syndromes. In contrast, in patients with active disease at alloSCT, with relapse being by far the most frequent cause of treatment failure, the classical risk factors such as cytogenetics, age and KPS determined outcome. Secondary AML had no influence on outcome in the context of the aggressive nature of advanced disease.

Patients diagnosed with sAML tend to be of older age, carry more unfavourable cytogenetics and show poor response to chemotherapy^20,21^. Regardless of cytogenetics and preceding therapy of antecedent haematological diseases, sAML is known to have a poor long-term remission even when using aggressive induction therapy^22,23^. Separate studies on patients with treatment-related AML following intensive chemotherapy, identified tAML as an independent adverse prognostic factor^2,7,14,24^. However, data on the role of sAML as a risk factor for outcome after alloSCT are scarce. Michelis et al. reported on a total of 264 patients with sAML and de novo AML, transplanted in CR1^12^. Age, hematopoietic cell transplant comorbidity index, and karyotype were considered for matching. In contrast to our results, no clear influence of having sAML could be demonstrated regarding OS, NRM, LFS, and RI after alloSCT, using multivariate analysis and a propensity score model. It can be speculated, that the lower patient numbers precluded Michelis at al. from detecting a statistically significant difference (*p* = 0.18), given that the 3-year OS in their cohort was 55% for de novo and 46% for sAML.

Besides the classical risk factors, i.e., cytogenetics and age, as well as donor type, our data identify sAML as an independent intrinsic factor for inferior outcome after alloSCT in CR1. Both patient and disease specific factors might contribute to this finding. First, characteristics associated with NRM (i.e., age, HLA mismatch, female donor to male patient, CMV seropositivity) had a high impact on OS in patients with sAML. This observation could suggest a lower ability to generally tolerate side effects and complications in the context of alloSCT, such as toxicity of the conditioning and immunosuppression, infections and GVHD. It might be a consequence of prior chemotherapy given for the antecedent hematologic or solid organ tumors, and might not be reflected by the performance score at time of alloSCT among patients with sAML^21,25^. Second, given the higher number of patients with sAML who have adverse cytogenetics, indicating lower sensitivity to standard chemotherapy and hypomethylating agents, the quality of CR might generally be inferior in sAML as compared to de novo disease. A better remission quality, however, is decisive for the outcome after alloSCT in sAML^26^, as well as de novo AML^27,28^. Third, the more aggressive nature of a secondary malignancy as such might play a role in the inferior outcome of patients with sAML, when transplanted in CR1.

Previous reports have suggested, that AML following antineoplastic radiation or chemotherapy (tAML) be considered an independent entity^25^. Although there are no pathognomonic genetic aberrations known for tAML, this subgroup had a worse outcome than MDS-related AML in several studies^2,7,14,24^. In a previous study from our group, slight differences in NRM and survival had been observed between patients with AML evolving from MDS/MPN and other hematologic malignancies, but not solid tumors^8^. In the present analysis, we therefore repeated the Cox model including tAML and AML evolving from another hematologic disease, as covariates. Both subgroups achieved comparable outcomes and were associated with increased OS, LFS and GRFS following alloSCT in CR1. Hence, as suggested earlier, the described two subtypes might be grouped as sAML in the setting of alloSCT^12^, although our numbers were to small to compare outcome of sAML following MDS versus sAML versus MPN.

Due to the retrospective nature of our study, some limitations, including missing information on MRD status and pre- and post-transplant therapies in most patients, must be acknowledged. In addition, there is a risk of selection bias, as patients without information on cytogenetics were deliberately excluded from this analysis, considering the high relevance of cytogenetic subgroups for outcome. Nevertheless, overall outcome data in our cohort are comparable to data from other studies^8^, suggesting that the cohort was representative. It must also be acknowledged that information on molecular aberrations was available only in a minority of patients from both cohorts and could therefore not be considered. However, in contrast to cytogenetic aberrations, which without doubt have similarly strong prognostic value in both de novo and sAML, less is known on the comparable value of molecular markers in both subgroups, which is why inclusion of these markers in this comparison would be difficult. Further, in the matched-pair analysis, patients with sAML and especially those in CR2 are most probably underrepresented, and fewer patients than expected could finally be included in the model. Therefore, the matched-pair analysis can only be regarded as confirmatory for the overall results obtained from the Cox model, which included all patients, and failed to reproduce the increased risk of relapse in sAML, most likely due to limited numbers.

This large-scale retrospective study identified sAML as an independent risk factor for alloSCT in CR1. Both the inferior quality of CR and higher sensitivity to toxicity might have contributed to the inferior outcome of sAML. These data help to improve risk stratification and prognostic estimates after alloSCT for sAML. Furthermore, the results may contribute to the design of optimized transplant protocols. Previous studies have suggested comparable outcomes after RIC and MAC in sAML, however, with a trend for better outcome after MAC^11,29^. Hence, a myeloablative, but reduced toxicity conditioning regimen such as the combination of Fludarabine and Treosulfan^30^ might be of particular value in patients with sAML. In addition, strategies to improve the quality of remission prior to alloSCT in those patients with frequently low sensitivity to chemotherapy might be of particular importance^31^.

## Supplementary information

Supplement
