# Supplementary material for: Inferior outcome of allogeneic stem cell transplantation for secondary acute myeloid leukemia in first complete remission as compared to de novo acute myeloid leukemia
Source: Blood Cancer J. 2020 Mar 3;10(3):26. doi: 10.1038/s41408-020-0296-3 (PMC7054545; doi:10.1038/s41408-020-0296-3)
Supplement: Supplementary file 1 — Supplement [file 41408_2020_296_MOESM1_ESM.docx]

**Table S1.** **Conditioning protocols in the entire population**

|  | **MAC** | **MAC** | **RIC** | **RIC** |
| --- | --- | --- | --- | --- |
|  | **de novo AML** | **sAML** | **de novo AML** | **sAML** |
| **BuCy*** | 2373 (37.68%) | 154 (26.83%) | 88 (1.71%) | 22 (2.93%) |
| **BuFlu*** | 1000 (15.88%) | 125 (21.78%) | 1969 (38.37%) | 296 (39.47%) |
| **TBF** | 358 (5.68%) | 28 (4.88%) | 153 (2.98%) | 22 (2.93%) |
| **FluMel** | 149 (2.37%) | 23 (4.01%) | 873 (17.01%) | 123 (16.4%) |
| **TreoFlu** | 231 (3.67%) | 53 (9.23%) | 163 (3.18%) | 42 (5.6%) |
| **FLAMSA** | 144 (2.29%) | 36 (6.27%) | 620 (12.08%) | 99 (13.2%) |
| **TBI** | 1884 (29.91%) | 140 (24.39%) | 1000 (19.49%) | 112 (14.93%) |
| **Other** | 159 (2.52%) | 15 (2.61%) | 266 (5.18%) | 34 (4.53%) |
| **Missing** | 8 | 0 | 1 | 1 |

MAC: myeloablative conditioning, RIC: reduced-intensity conditioning, Bu: Busulfan, Cy: Cyclophosphomide, Flu: Fludarabine, Mel: Melphalan, Treo: Treosulfan, TBF: Thiothepa/Busulfan/Fludarabine, TBI: Total Body Irradiation

***** According to the EBMT definition, regimen containing IV Busulfan >=6.4 mg/kg or oral Busulfan >=8 mg/kg are considered as RIC

**Table S2.** **Immunosuppression**

|  | **de novo AML** | **sAML** |
| --- | --- | --- |
| CSA | 1913 (16.81%) | 212 (16.02%) |
| CSA+MTX | 5221 (45.89%) | 464 (35.07%) |
| CSA+MMF | 2509 (22.05%) | 422 (31.9%) |
| CSA+MTX+MMF | 133 (1.17%) | 18 (1.36%) |
| TAC+SIRO | 158 (1.39%) | 9 (0.68%) |
| MMF+TAC | 255 (2.24%) | 28 (2.12%) |
| MTX+TAC | 165 (1.45%) | 33 (2.49%) |
| PTCY | 677 (5.95%) | 89 (6.73%) |
| Other | 347 (3.05%) | 48 (3.63%) |
| Missing | 61 | 2 |

CSA: Cyclosporin A, MTX: Methotrexate, MMF: Mycophenolate mofetil, TAC: tacrolimus, SIRO: sirolimus, PTCY: post-transplant cyclophosphamide

**Table S3.** **Causes of death in the entire population**

| **Causes of death** | **de novo AML** | **sAML** |
| --- | --- | --- |
| **N** | 9607 | 1772 |
| Cardiac toxicity | 29 (0.6%) | 7 (1%) |
| Haemorhage | 80 (1.67%) | 10 (1.43%) |
| Failure/Rejection | 17 (0.35%) | 2 (0.29%) |
| Veno-occlusive disease | 84 (1.75%) | 9 (1.29%) |
| Infection | 848 (17.67%) | 147 (21.03%) |
| Interstitial pneumonitis | 104 (2.17%) | 14 (2%) |
| GVHD | 833 (17.36%) | 93 (13.3%) |
| Original disease | 2461 (51.28%) | 357 (51.07%) |
| Second malignancy | 80 (1.67%) | 13 (1.86%) |
| Other transplant related | 263 (5.48%) | 47 (6.72%) |
| NA | 227 | 50 |

**Table S4.** **Multivariate analysis of risk factors for cumulative incidence of relapse, stratified by stage at transplantation**

|  | **SCT in CR1** | | | **SCT in PIF** | | | **SCT in Relapse** | | |
| --- | --- | --- | --- | --- | --- | --- | --- | --- | --- |
|  | **HR** | **CI** | **p** | **HR** | **CI** | **p** | **HR** | **CI** | **p** |
| **sAML** | 1.27 | 1.12 - 1.44 | **<10^-3^** | 0.932 | 0.705 - 1.232 | 0.621 | 0.878 | 0.661 - 1.166 | 0.368 |
| **age (per 10 years)** | 1.014 | 0.976 - 1.053 | 0.471 | 0.974 | 0.895 - 1.059 | 0.533 | 0.945 | 0.887 - 1.008 | 0.087 |
| **year of SCT** | 1.009 | 0.998 - 1.02 | 0.114 | 0.959 | 0.933 - 0.984 | **0.002** | 0.991 | 0.97 - 1.012 | 0.402 |
| **relapse 2 vs relapse 1** | n.a. |  |  | n.a. |  |  | 1.117 | 0.898 - 1.389 | 0.32 |
| **favorable cytogenetics (ref)** | 1 |  |  | 1 |  |  | 1 |  |  |
| **intermediate** | 1.247 | 1.04 - 1.495 | **0.017** | n.a. | n.a. |  | n.a. |  |  |
| **adverse** | 2.34 | 1.936 - 2.828 | **<10^-5^** | 1.521 | 1.224 - 1.889 | **<10^-3^** | 1.563 | 1.293 - 1.888 | **<10^-5^** |
| **female donor to male recipient** | 0.945 | 0.85 - 1.051 | 0.297 | 1.007 | 0.779 - 1.302 | 0.955 | 1.126 | 0.92 - 1.377 | 0.248 |
| **Previous autograft** | 0.9 | 0.58 - 1.394 | 0.636 | n.a. |  |  | n.a. |  |  |
| **KPS>80%** | 0.866 | 0.69 - 1.086 | 0.213 | 0.644 | 0.474 - 0.876 | **0.005** | 0.679 | 0.537 - 0.858 | **0.001** |
| **MSD (ref)** | 1 |  |  | 1 |  |  | 1 |  |  |
| **UD 10/10** | 0.82 | 0.736 - 0.914 | **<10^-4^** | 0.807 | 0.598 - 1.087 | 0.158 | 0.825 | 0.665 - 1.024 | 0.081 |
| **UD 9/10** | 0.871 | 0.736 - 1.029 | 0.104 | 0.735 | 0.493 - 1.096 | 0.131 | 0.974 | 0.74 - 1.281 | 0.848 |
| **Haploidentical donor** | 0.723 | 0.564 - 0.926 | **0.01** | 1.226 | 0.804 - 1.87 | 0.345 | 0.724 | 0.529 - 0.993 | **0.045** |
| **PB vs BM** | 0.975 | 0.866 - 1.097 | 0.671 | 0.983 | 0.676 - 1.429 | 0.929 | 0.904 | 0.692 - 1.181 | 0.459 |
| **Patient CMV positive** | 0.994 | 0.907 - 1.09 | 0.9 | 1.013 | 0.8 - 1.283 | 0.915 | 1.04 | 0.862 - 1.256 | 0.681 |
| **Donor CMV positive** | 0.953 | 0.872 - 1.041 | 0.283 | 1.02 | 0.815 - 1.278 | 0.86 | 1.137 | 0.954 - 1.355 | 0.152 |
| **MAC vs RIC** | 0.838 | 0.755 - 0.93 | **0.001** | 0.97 | 0.766 - 1.23 | 0.803 | 0.958 | 0.796 - 1.154 | 0.654 |
| **in vivo T-cell depletion** | 1.112 | 1.007 - 1.229 | **0.036** | 1.065 | 0.813 - 1.394 | 0.648 | 1.064 | 0.873 - 1.296 | 0.539 |

SCT: stem cell transplantation, CR: complete remission, PIF: primary induction failure, ref: reference, KPS: Karnofsky performance status, MSD: matched sibling donor, UD: unrelated donor, PB: peripheral blood, BM: bone marrow, CMV: cytomegalovirus, MAC: myeloablative conditioning, RIC: reduced-intensity conditioning

**Table S5. Multivariate analysis of risk factors for non-relapse mortality, stratified by stage at transplantation**

|  | **SCT in CR1** | | | **SCT in PIF** | | | **SCT in Relapse** | | |
| --- | --- | --- | --- | --- | --- | --- | --- | --- | --- |
|  | **HR** | **CI** | **p** | **HR** | **CI** | **p** | **HR** | **CI** | **p** |
| **sAML** | 1.37 | 1.17 - 1.59 | **<10^-4^** | 1.091 | 0.762 - 1.563 | 0.634 | 1.182 | 0.821 - 1.701 | 0.37 |
| **age (per 10 years)** | 1.357 | 1.288 - 1.431 | **<10^-5^** | 1.243 | 1.093 - 1.413 | **0.001** | 1.224 | 1.105 - 1.356 | **<10^-4^** |
| **year of SCT** | 0.983 | 0.968 - 0.998 | **0.027** | 1.005 | 0.965 - 1.045 | 0.823 | 1.009 | 0.976 - 1.042 | 0.612 |
| **relapse 2 vs relapse 1** | n.a. |  |  | n.a. |  |  | 1.557 | 1.163 - 2.085 | **0.003** |
| **favorable cytogenetics (ref)** | 1 |  |  | 1 |  |  | 1 |  |  |
| **intermediate** | 1.099 | 0.885 - 1.363 | 0.394 | n.a. | n.a. |  | n.a. |  |  |
| **adverse** | 1.193 | 0.941 - 1.513 | 0.144 | 1.529 | 1.122 - 2.083 | **0.007** | 1.157 | 0.857 - 1.562 | 0.342 |
| **female donor to male recipient** | 1.342 | 1.185 - 1.519 | **<10^-5^** | 1.343 | 0.938 - 1.922 | 0.108 | 1.117 | 0.821 - 1.521 | 0.482 |
| **Previous autograft** | 1.852 | 1.23 - 2.79 | **0.003** | n.a. |  |  | n.a. |  |  |
| **KPS>80%** | 0.551 | 0.427 - 0.711 | **<10^-5^** | 0.511 | 0.349 - 0.749 | **0.001** | 0.558 | 0.411 - 0.758 | **<10^-4^** |
| **MSD (ref)** | 1 |  |  | 1 |  |  | 1 |  |  |
| **UD 10/10** | 1.587 | 1.377 - 1.829 | **<10^-5^** | 1.311 | 0.861 - 1.998 | 0.207 | 0.817 | 0.586 - 1.138 | 0.231 |
| **UD 9/10** | 2.15 | 1.758 - 2.629 | **<10^-5^** | 1.396 | 0.832 - 2.343 | 0.206 | 1.342 | 0.912 - 1.976 | 0.136 |
| **Haploidentical donor** | 2.35 | 1.837 - 3.007 | **<10^-5^** | 1.221 | 0.673 - 2.214 | 0.512 | 1.114 | 0.737 - 1.684 | 0.609 |
| **PB vs BM** | 1.011 | 0.867 - 1.179 | 0.887 | 0.858 | 0.497 - 1.481 | 0.583 | 0.665 | 0.467 - 0.945 | **0.023** |
| **Patient CMV positive** | 1.206 | 1.067 - 1.363 | **0.003** | 1.577 | 1.102 - 2.258 | **0.013** | 1.03 | 0.772 - 1.374 | 0.841 |
| **Donor CMV positive** | 1.015 | 0.906 - 1.137 | 0.797 | 0.964 | 0.698 - 1.332 | 0.825 | 1.16 | 0.893 - 1.506 | 0.267 |
| **MAC vs RIC** | 1.34 | 1.171 - 1.534 | **<10^-4^** | 1.04 | 0.749 - 1.445 | 0.813 | 1.012 | 0.782 - 1.311 | 0.926 |
| **in vivo T-cell depletion** | 0.8 | 0.701 - 0.914 | **0.001** | 0.819 | 0.562 - 1.194 | 0.299 | 1.095 | 0.826 - 1.451 | 0.527 |

SCT: stem cell transplantation, CR: complete remission, PIF: primary induction failure, ref: reference, KPS: Karnofsky performance status, MSD: matched sibling donor, UD: unrelated donor, PB: peripheral blood, BM: bone marrow, CMV: cytomegalovirus, MAC: myeloablative conditioning, RIC: reduced-intensity conditioning

**Table S6.** **Multivariate analysis of risk factors for leukemia-free survival, stratified by stage at transplantation**

|  | **SCT in CR1** | | | **SCT in PIF** | | | **SCT in Relapse** | | |
| --- | --- | --- | --- | --- | --- | --- | --- | --- | --- |
|  | **HR** | **CI** | **p** | **HR** | **CI** | **p** | **HR** | **CI** | **p** |
| **sAML** | 1.32 | 1.19 - 1.45 | **<10^-5^** | 0.983 | 0.787 - 1.229 | 0.881 | 0.961 | 0.767 - 1.205 | 0.732 |
| **age (per 10 years)** | 1.126 | 1.092 - 1.161 | **<10^-5^** | 1.054 | 0.982 - 1.13 | 0.143 | 1.02 | 0.966 - 1.077 | 0.47 |
| **year of SCT** | 1.001 | 0.992 - 1.01 | 0.863 | 0.971 | 0.95 - 0.993 | **0.01** | 0.995 | 0.977 - 1.012 | 0.554 |
| **relapse 2 vs relapse 1** | n.a. |  |  | n.a. |  |  | 1.239 | 1.04 - 1.476 | **0.017** |
| **favorable cytogenetics (ref)** | 1 |  |  | 1 |  |  | 1 |  |  |
| **intermediate** | 1.182 | 1.029 - 1.358 | **0.018** | n.a. | n.a. |  | n.a. |  |  |
| **adverse** | 1.83 | 1.58 - 2.12 | **<10^-5^** | 1.528 | 1.279 - 1.825 | **<10^-5^** | 1.438 | 1.225 - 1.688 | **10^-5^** |
| **female donor to male recipient** | 1.086 | 1.002 - 1.176 | **0.045** | 1.113 | 0.903 - 1.372 | 0.313 | 1.127 | 0.952 - 1.334 | 0.167 |
| **Previous autograft** | 1.253 | 0.932 - 1.684 | 0.136 | n.a. |  |  | n.a. |  |  |
| **KPS>80%** | 0.722 | 0.61 - 0.855 | **<10-3** | 0.586 | 0.461 - 0.746 | **10^-5^** | 0.632 | 0.524 - 0.763 | **<10^-5^** |
| **MSD (ref)** | 1 |  |  | 1 |  |  | 1 |  |  |
| **UD 10/10** | 1.041 | 0.956 - 1.134 | 0.357 | 0.955 | 0.749 - 1.217 | 0.709 | 0.832 | 0.694 - 0.997 | **0.047** |
| **UD 9/10** | 1.209 | 1.064 - 1.373 | **0.004** | 0.923 | 0.673 - 1.266 | 0.621 | 1.072 | 0.857 - 1.342 | 0.541 |
| **Haploidentical donor** | 1.185 | 0.999 - 1.405 | 0.051 | 1.227 | 0.868 - 1.735 | 0.246 | 0.863 | 0.67 - 1.113 | 0.258 |
| **PB vs BM** | 0.99 | 0.901 - 1.086 | 0.825 | 0.919 | 0.674 - 1.252 | 0.591 | 0.825 | 0.665 - 1.022 | 0.079 |
| **Patient CMV positive** | 1.067 | 0.992 - 1.149 | 0.082 | 1.163 | 0.956 - 1.416 | 0.132 | 1.037 | 0.885 - 1.214 | 0.654 |
| **Donor CMV positive** | 0.973 | 0.907 - 1.043 | 0.435 | 1.005 | 0.835 - 1.21 | 0.956 | 1.142 | 0.986 - 1.321 | 0.076 |
| **MAC vs RIC** | 0.995 | 0.917 - 1.08 | 0.906 | 0.989 | 0.815 - 1.201 | 0.915 | 0.991 | 0.849 - 1.156 | 0.909 |
| **in vivo T-cell depletion** | 0.987 | 0.912 - 1.068 | 0.746 | 0.965 | 0.775 - 1.202 | 0.752 | 1.068 | 0.907 - 1.258 | 0.431 |

SCT: stem cell transplantation, CR: complete remission, PIF: primary induction failure, ref: reference, KPS: Karnofsky performance status, MSD: matched sibling donor, UD: unrelated donor, PB: peripheral blood, BM: bone marrow, CMV: cytomegalovirus, MAC: myeloablative conditioning, RIC: reduced-intensity conditioning

**Table S7.** **Multivariate analysis of risk factors for GVHD/Relapse-free survival, stratified by stage at transplantation**

|  | **SCT in CR1** | | | **SCT in PIF** | | | **SCT in Relapse** | | |
| --- | --- | --- | --- | --- | --- | --- | --- | --- | --- |
|  | **HR** | **CI** | **p** | **HR** | **CI** | **p** | **HR** | **CI** | **p** |
| **sAML** | 1.19 | 1.093 - 1.307 | **<10^-4^** | 1.013 | 0.829 - 1.237 | 0.9 | 0.971 | 0.778 - 1.21 | 0.791 |
| **age (per 10 years)** | 1.087 | 1.059 - 1.117 | **<10^-5^** | 1.02 | 0.957 - 1.088 | 0.541 | 0.993 | 0.942 - 1.048 | 0.806 |
| **year of SCT** | 0.997 | 0.989 - 1.005 | 0.455 | 0.988 | 0.968 - 1.008 | 0.239 | 0.989 | 0.972 - 1.007 | 0.227 |
| **relapse 2 vs relapse 1** | n.a. |  |  | n.a. |  |  | 1.116 | 0.939 - 1.326 | 0.214 |
| **favorable cytogenetics (ref)** | 1 |  |  | 1 |  |  | 1 |  |  |
| **intermediate** | 1.183 | 1.05 - 1.333 | **0.006** | n.a. | n.a. |  | n.a. |  |  |
| **adverse** | 1.641 | 1.444 - 1.866 | **<10^-5^** | 1.416 | 1.203 - 1.667 | **<10^-4^** | 1.341 | 1.146 - 1.569 | **<10^-3^** |
| **female donor to male recipient** | 1.228 | 1.145 - 1.316 | **<10^-5^** | 1.166 | 0.961 - 1.414 | 0.12 | 1.136 | 0.963 - 1.341 | 0.131 |
| **Previous autograft** | 1.219 | 0.923 - 1.609 | 0.164 | n.a. |  |  | n.a. |  |  |
| **KPS>80%** | 0.788 | 0.673 - 0.924 | **0.003** | 0.591 | 0.475 - 0.737 | **<10^-5^** | 0.695 | 0.578 - 0.837 | **<10^-3^** |
| **MSD (ref)** | 1 |  |  | 1 |  |  | 1 |  |  |
| **UD 10/10** | 1.071 | 0.992 - 1.155 | 0.078 | 1.154 | 0.924 - 1.442 | 0.207 | 0.883 | 0.74 - 1.054 | 0.168 |
| **UD 9/10** | 1.241 | 1.106 - 1.392 | **<10^-4^** | 1.091 | 0.816 - 1.459 | 0.555 | 1.101 | 0.881 - 1.376 | 0.397 |
| **Haploidentical donor** | 1.039 | 0.89 - 1.212 | 0.63 | 1.111 | 0.807 - 1.531 | 0.519 | 0.939 | 0.735 - 1.2 | 0.616 |
| **PB vs BM** | 1.121 | 1.031 - 1.219 | **0.007** | 1.175 | 0.879 - 1.572 | 0.277 | 0.968 | 0.784 - 1.194 | 0.759 |
| **Patient CMV positive** | 1.03 | 0.966 - 1.099 | 0.367 | 1.095 | 0.912 - 1.314 | 0.33 | 1.046 | 0.895 - 1.222 | 0.572 |
| **Donor CMV positive** | 0.999 | 0.939 - 1.062 | 0.968 | 0.935 | 0.788 - 1.11 | 0.444 | 1.182 | 1.024 - 1.365 | **0.023** |
| **MAC vs RIC** | 0.978 | 0.909 - 1.052 | 0.547 | 0.921 | 0.772 - 1.099 | 0.361 | 0.941 | 0.81 - 1.095 | 0.433 |
| **in vivo T-cell depletion** | 0.8 | 0.745 - 0.858 | **<10^-5^** | 0.679 | 0.554 - 0.832 | **<10^-4^** | 0.931 | 0.792 - 1.094 | 0.384 |

SCT: stem cell transplantation, CR: complete remission, PIF: primary induction failure, ref: reference, KPS: Karnofsky performance status, MSD: matched sibling donor, UD: unrelated donor, PB: peripheral blood, BM: bone marrow, CMV: cytomegalovirus, MAC: myeloablative conditioning, RIC: reduced-intensity conditioning

**Table S8**. **Separate comparison between de novo AML and AML evolving from myeloid malignancies or bone marrow failure syndromes,**

**and between de novo AML and treatment-related AML**

|  | **de novo AML**  **(n=7691; reference)** | **AML secondary**  **to myeloid malignancies**  **(n=537)** | | | **treatment-related AML**  **(n=372)** | | | |
| --- | --- | --- | --- | --- | --- | --- | --- | --- |
|  | **3 year outcome** | **3 year outcome** | **HR** | **p (Cox)** | **3 year outcome** | **HR** | **p (Cox)** |  |
| **Relapse** | 28.5% | 37.1% | 1.36 | <0.0001 | 31.6% | 1.15 | 0.15 |  |
| **NRM** | 16.4% | 22.5% | 1.23 | 0.04 | 24.8% | 1.58 | <0.0001 |  |
| **OS** | 60.8% | 47.3% | 1.29 | 0.0001 | 46.4% | 1.4 | <0.0001 |  |
| **LFS** | 55.1% | 40.4% | 1.31 | <0.0001 | 43.6% | 1.32 | <0.0001 |  |
| **GRFS** | 38.6% | 28.4% | 1.19 | 0.0002 | 28.7% | 1.22 | 0.0003 |  |

BM: bone marrow, HR: hazard ratio, OS: overall survival, NRM: non-relapse mortality, LFS: leukemia-free survival, GRFS: Graft-versus-Host Disease/relapse-free survival

**Table S9.** **Multivariate analysis of risk factors for acute GVHD II-IV, stratified by stage at transplantation**

|  | **SCT in CR1** | | | **SCT in PIF** | | | **SCT in Relapse** | | |
| --- | --- | --- | --- | --- | --- | --- | --- | --- | --- |
|  | **HR** | **CI** | **p** | **HR** | **CI** | **p** | **HR** | **CI** | **p** |
| **sAML** | 1.144 | 0.981 - 1.334 | 0.086 | 0.736 | 0.504 - 1.074 | 0.112 | 1.152 | 0.776 - 1.711 | 0.484 |
| **age (per 10 years)** | 1.05 | 1.005 - 1.097 | **0.03** | 0.935 | 0.839 - 1.041 | 0.22 | 0.915 | 0.834 - 1.005 | 0.064 |
| **year of SCT** | 0.982 | 0.97 - 0.994 | **0.004** | 1 | 0.966 - 1.036 | 0.983 | 0.99 | 0.96 - 1.02 | 0.502 |
| **relapse 2 vs relapse 1** | n.a. |  |  | n.a. |  |  | 1.1 | 0.809 - 1.494 | 0.543 |
| **favorable cytogenetics (ref)** | 1 |  |  | 1 |  |  | 1 |  |  |
| **intermediate** | 1.539 | 1.233 - 1.922 | **<10^-3^** | n.a. | n.a. |  | n.a. |  |  |
| **adverse** | 1.532 | 1.208 - 1.943 | **<10^-3^** | 1.611 | 1.213 - 2.139 | **0.001** | 0.895 | 0.67 - 1.195 | 0.452 |
| **female donor to male recipient** | 1.237 | 1.103 - 1.387 | **<10^-3^** | 1.532 | 1.121 - 2.093 | **0.007** | 1.378 | 1.042 - 1.824 | **0.025** |
| **Previous autograft** | 0.802 | 0.466 - 1.379 | 0.424 | n.a. |  |  | n.a. |  |  |
| **KPS>80%** | 0.705 | 0.547 - 0.91 | **0.007** | 0.738 | 0.504 - 1.082 | 0.119 | 1.005 | 0.712 - 1.418 | 0.979 |
| **MSD (ref)** | 1 |  |  | 1 |  |  | 1 |  |  |
| **UD 10/10** | 1.661 | 1.464 - 1.883 | **<10^-5^** | 2.037 | 1.385 - 2.995 | **<10^-3^** | 1.396 | 1.012 - 1.926 | **0.042** |
| **UD 9/10** | 2.203 | 1.842 - 2.635 | **<10^-5^** | 2.389 | 1.485 - 3.841 | **<10^-3^** | 1.838 | 1.259 - 2.683 | **0.001** |
| **Haploidentical donor** | 1.679 | 1.334 - 2.113 | **10^-5^** | 1.354 | 0.787 - 2.33 | 0.273 | 1.25 | 0.82 - 1.905 | 0.3 |
| **PB vs BM** | 1.049 | 0.916 - 1.201 | 0.488 | 1.652 | 0.961 - 2.839 | 0.069 | 1.373 | 0.922 - 2.046 | 0.119 |
| **Patient CMV positive** | 0.884 | 0.795 - 0.983 | **0.023** | 0.778 | 0.573 - 1.057 | 0.108 | 0.855 | 0.656 - 1.112 | 0.243 |
| **Donor CMV positive** | 1.011 | 0.912 - 1.12 | 0.836 | 1.114 | 0.825 - 1.506 | 0.481 | 1.089 | 0.849 - 1.397 | 0.503 |
| **MAC vs RIC** | 1.383 | 1.222 - 1.564 | **<10^-5^** | 1.095 | 0.804 - 1.491 | 0.566 | 0.976 | 0.752 - 1.268 | 0.857 |
| **in vivo T-cell depletion** | 0.637 | 0.563 - 0.721 | **<10^-5^** | 0.524 | 0.37 - 0.744 | **<10^-3^** | 0.799 | 0.603 - 1.058 | 0.118 |

SCT: stem cell transplantation, CR: complete remission, PIF: primary induction failure, ref: reference, KPS: Karnofsky performance status, MSD: matched sibling donor, UD: unrelated donor, PB: peripheral blood, BM: bone marrow, CMV: cytomegalovirus, MAC: myeloablative conditioning, RIC: reduced-intensity conditioning

**Table S10.** **Multivariate analysis of risk factors for chronic GVHD, stratified by stage at transplantation**

|  |  | **SCT in CR1** |  |  | **SCT in PIF** |  |  | **SCT in Relapse** |  |
| --- | --- | --- | --- | --- | --- | --- | --- | --- | --- |
|  | **HR** | **CI** | **p** | **HR** | **CI** | **p** | **HR** | **CI** | **p** |
| **sAML** | 1.039 | 0.915 - 1.179 | 0.556 | 0.965 | 0.672 - 1.385 | 0.845 | 0.879 | 0.58 - 1.331 | 0.542 |
| **age (per 10 years)** | 1.061 | 1.026 - 1.098 | **0.001** | 1.019 | 0.904 - 1.149 | 0.753 | 1.04 | 0.942 - 1.147 | 0.437 |
| **year of SCT** | 0.993 | 0.983 - 1.002 | 0.132 | 0.98 | 0.945 - 1.016 | 0.277 | 0.953 | 0.922 - 0.984 | **0.003** |
| **relapse 2 vs relapse 1** | n.a. |  |  | n.a. |  |  | 0.877 | 0.625 - 1.231 | 0.448 |
| **favorable cytogenetics (ref)** | 1 |  |  | 1 |  |  | 1 |  |  |
| **intermediate** | 1.008 | 0.878 - 1.158 | 0.907 | n.a. | n.a. |  | n.a. |  |  |
| **adverse** | 1.037 | 0.887 - 1.212 | 0.651 | 1.042 | 0.769 - 1.411 | 0.792 | 1.259 | 0.926 - 1.713 | 0.142 |
| **female donor to male recipient** | 1.367 | 1.254 - 1.49 | **<10^-5^** | 1.625 | 1.165 - 2.266 | **0.004** | 1.042 | 0.767 - 1.416 | 0.791 |
| **Previous autograft** | 1.336 | 0.906 - 1.97 | 0.144 | n.a. |  |  | n.a. |  |  |
| **KPS>80%** | 0.776 | 0.623 - 0.968 | **0.024** | 1.216 | 0.747 - 1.979 | 0.431 | 0.796 | 0.554 - 1.144 | 0.217 |
| **MSD (ref)** | 1 |  |  | 1 |  |  | 1 |  |  |
| **UD 10/10** | 1.178 | 1.067 - 1.301 | **0.001** | 1.307 | 0.879 - 1.943 | 0.186 | 0.734 | 0.531 - 1.013 | 0.06 |
| **UD 9/10** | 1.326 | 1.138 - 1.546 | **<10^-3^** | 1.045 | 0.634 - 1.723 | 0.862 | 0.93 | 0.606 - 1.428 | 0.741 |
| **Haploidentical donor** | 0.808 | 0.649 - 1.006 | 0.057 | 0.611 | 0.3 - 1.244 | 0.175 | 0.938 | 0.589 - 1.494 | 0.788 |
| **PB vs BM** | 1.371 | 1.226 - 1.534 | **<10^-5^** | 2.683 | 1.305 - 5.518 | **0.007** | 2.066 | 1.261 - 3.386 | **0.004** |
| **Patient CMV positive** | 0.975 | 0.897 - 1.059 | 0.543 | 1.225 | 0.89 - 1.687 | 0.214 | 1.149 | 0.869 - 1.52 | 0.33 |
| **Donor CMV positive** | 1.059 | 0.978 - 1.147 | 0.155 | 0.869 | 0.639 - 1.182 | 0.372 | 0.965 | 0.743 - 1.252 | 0.786 |
| **MAC vs RIC** | 1.03 | 0.935 - 1.134 | 0.553 | 0.786 | 0.565 - 1.094 | 0.154 | 0.906 | 0.687 - 1.195 | 0.484 |
| **in vivo T-cell depletion** | 0.585 | 0.531 - 0.645 | **<10^-5^** | 0.474 | 0.327 - 0.686 | **<10^-4^** | 0.69 | 0.515 - 0.925 | **0.013** |

SCT: stem cell transplantation, CR: complete remission, PIF: primary induction failure, ref: reference, KPS: Karnofsky performance status, MSD: matched sibling donor, UD: unrelated donor, PB: peripheral blood, BM: bone marrow, CMV: cytomegalovirus, MAC: myeloablative conditioning, RIC: reduced-intensity conditioning

**Table S11. Results of pair-matching analysis among patients with de novo und secondary AML**

| **Criteria** | **de novo AML** | **sAML** | **P** |
| --- | --- | --- | --- |
| **N** | 877 | 877 |  |
| median follow-up (months) (range) (IQR) | 25.5 (1-212.2) (11.5-63.9) | 37.4 (1.8-191.3) (14.8-66.4) | 0.005 |
| Age at SCT (year), median (range) (IQR) | 57.2 (18.2-76.1) (49.4-62.8) | 57.4 (18.3-74.6) (49.5-62.9) | 0.732 |
| Year of SCT, median (range) (IQR) | 2011 (2000-2016) (2008-2015) | 2012 (2000-2016) (2009-2014) | 0.603 |
| Months diagnosis to SCT, median (IQR) | 5.2 (4-6.8) | 4.6 (3.5-6.2) | <10^-3^ |
| **Status at SCT*** |  |  |  |
| CR1 | 719 (81.98%) | 719 (81.98%) | 1 |
| CR2 | 47 (5.36%) | 47 (5.36%) |  |
| PIF | 67 (7.64%) | 67 (7.64%) |  |
| Relapse | 44 (5.02%) | 44 (5.02%) |  |
| **Sex patient** |  |  |  |
| Male | 466 (53.14%) | 414 (47.21%) | 0.013 |
| Female | 411 (46.86%) | 463 (52.79%) |  |
| **Sex donor** |  |  |  |
| Male | 590 (67.43%) | 550 (62.71%) | 0.038 |
| Female | 285 (32.57%) | 327 (37.29%) |  |
| **Donor to patient sex*** |  |  |  |
| No female to male | 767 (87.46%) | 767 (87.46%) | 1 |
| Female to male | 110 (12.54%) | 110 (12.54%) |  |
| **KPS at SCT*** |  |  |  |
| KPS<80 | 15 (1.71%) | 15 (1.71%) | 1 |
| KPS>=80 | 862 (98.29%) | 862 (98.29%) |  |
| **Donor type*** |  |  |  |
| MSD | 431 (49.14%) | 431 (49.14%) | 1 |
| UD 10/10 | 360 (41.05%) | 360 (41.05%) |  |
| UD 9/10 | 58 (6.61%) | 58 (6.61%) |  |
| Haploidentical donor | 28 (3.19%) | 28 (3.19%) |  |
| **CMV status patient** |  |  |  |
| Pat. CMV negative | 255 (29.08%) | 255 (29.08%) | 1 |
| Pat. CMV positive | 622 (70.92%) | 622 (70.92%) |  |
| **CMV status donor** |  |  |  |
| Donor CMV negative | 428 (48.8%) | 428 (48.8%) | 1 |
| Donor CMV positive | 449 (51.2%) | 449 (51.2%) |  |
| **CMV status donor (D) and recipient (R)*** |  |  |  |
| D-/R- | 196 (22.35%) | 196 (22.35%) | 1 |
| D+/R- | 59 (6.73%) | 59 (6.73%) |  |
| D-/R+ | 232 (26.45%) | 232 (26.45%) |  |
| D+/R+ | 390 (44.47%) | 390 (44.47%) |  |
| **Cytogenetics*** |  |  |  |
| Favorable | 29 (3.31%) | 29 (3.31%) | 1 |
| Intermediate | 626 (71.38%) | 626 (71.38%) |  |
| Adverse | 222 (25.31%) | 222 (25.31%) |  |
| **Graft source*** |  |  |  |
| Bone marrow | 68 (7.75%) | 68 (7.75%) | 1 |
| Peripheral blood | 809 (92.25%) | 809 (92.25%) |  |
| **Conditioning regimen*** |  |  |  |
| MAC | 365 (41.62%) | 365 (41.62%) | 1 |
| RIC | 512 (58.38%) | 512 (58.38%) |  |
| **T-cell depletion (TCD)*** |  |  |  |
| No in vivo TCD | 319 (36.37%) | 319 (36.37%) | 1 |
| In vivo TCD | 558 (63.63%) | 558 (63.63%) |  |
| No ex vivo TCD | 873 (99.54%) | 873 (99.54%) | 1 |
| Ex vivo TCD | 4 (0.46%) | 4 (0.46%) |  |

* denotes matching criteria

Graft source: 2 missing in de novo AML group

IQR: interquartile range, SCT: stem cell transplantation, CR: complete remission, PIF: primary induction failure, KPS: Karnofsky performance status, MSD: matched sibling donor, UD: unrelated donor, CMV: cytomegalovirus, MAC: myeloablative conditioning, RIC: reduced-intensity conditioning, TCD: T-cell depletion

**Table S12.** **Data were contributed by the following EBMT centers:**

| **Center** |
| --- |
|  |
| 207 Paris [St Louis] |
| 515 Helsinki [Univ Central H] |
| 230 Marseille [Paoli Calmettes] |
| 277 Lille [H Claude Huriez] |
| 267 Pessac [H Haut-Leveque] |
| 246 Rotterdam [Erasmus MC] |
| 295 Hannover [Medical Univ] |
| 259 Essen [Univ H] |
| 671 Lyon [H E Herriot] |
| 202 Basel [202] |
| 624 Toulouse [H Purpan] |
| 209 Leuven [Univ H] |
| 387 Birmingham [Queen Elizabeth] |
| 672 Strasbourg [H Hautepierre] |
| 810 Freiburg [University] |
| 253 Nantes [Hotel Dieu] |
| 926 Montpellier [University] |
| 235 Oslo [Rikshospitalet] |
| 397 Riyadh [King Faisal] |
| 717 Nottingham [City H] |
| 813 Milano [S Raffaele] |
| 270 Grenoble [H A Michallon] |
| 633 Teheran [Shariati] |
| 311 Wiesbaden [Kl Diagnostik] |
| 556 Budapest [National Med Ctr] |
| 756 Rome [Tor Vergata] |
| 212 Stockholm [Univ H] |
| 204 Ulm [Innere Med III] |
| 614 Hamburg [Univ H] |
| 565 Maastricht [Univ H] |
| 676 Vandoeuvre_Les_Nancy [H d`Enfants] |
| 237 Nijmegen [St Radboud] |
| 666 Villejuif [Gustave Roussy] |
| 650 Angers [CHRU] |
| 225 Turku [University] |
| 234 Brussels [St. Luc] |
| 546 Groningen [Univ H] |
| 242 Santander [Valdecilla] |
| 718 Pilsen [Charles Univ H] |
| 283 Lund [Univ H] |
| 680 Muenster [University] |
| 513 Munich [Kl Grosshadern] |
| 566 Cambridge [Addenbrookes H] |
| 661 Rennes [H Sud/Pontchaillou] |
| 775 Paris [St Antoine] |
| 203 Leiden [Univ H] |
| 726 Liege [University] |
| 252 Creteil [H Mondor Hematol] |
| 251 Caen [Hopital, Hematol] |
| 754 Tel-Hashomer [Univ Adults] |
| 808 Dresden [Universitaets Kl] |
| 785 Homburg [Univ Saarland] |
| 264 Poitiers [H La Miletrie] |
| 941 Rouen [Becquerel] |
| 727 Salamanca [H Clinico] |
| 260 Barcelona [SCreu i S Pau] |
| 996 Antwerp_Edegem [UZA] |
| 224 London [UCL] |
| 659 Brest [C.H.R.U Brest] |
| 658 Bergamo [Ospedale, ematol] |
| 704 Southampton [General H] |
| 206 Copenhagen [Rigshospitalet] |
| 239 Utrecht [University] |
| 152 Augsburg [Zentral Kl] |
| 731 Umeå [Univ H] |
| 544 Monza [Osp S Gerardo] |
| 261 Geneva [261] |
| 273 Clermont-Ferrand [Jean Perrin] |
| 389 Leipzig [Univ, Haemat/Oncol] |
| 524 Heidelberg [Medizinische Kl] |
| 778 Sheffield [Royal Hallamshire] |
| 588 Amsterdam [VU Univ Med Ctr] |
| 625 Nuernberg [Klinikum] |
| 597 Brno [Univ H] |
| 725 St._Petersburg [Pavlov Med Univ] |
| 248 Pescara [Osp Civile] |
| 215 Brussels [Jules Bordet] |
| 534 Cologne [Univ, Medicine] |
| 250 Saint_Etienne [St Etienne] |
| 713 Leicester [Royal Infirmary] |
| 145 Stuttgart [Robert_Bosch_Kh] |
| 218 London [Royal Marsden] |
| 613 Barcelona [H Trias i Pujol] |
| 977 Limoges [CHRU] |
| 231 Torino [S. Giovanni (CTO)] |
| 169 Ankara [Gazi Univ] |
| 523 Nice [H de l`ARCHET I] |
| 665 Clamart [H Percy] |
| 257 Dublin [St James] |
| 692 Palermo [La Maddalena] |
| 994 Istanbul [Nightingale] |
| 561 Thessaloniki [Papanicolaou G H] |
| 759 Barcelona [H Univ Bellvitge] |
| 214 Barcelona [H Clinic] |
| 223 Tuebingen [Univ] |
| 232 Rome [Emat, La Sapienza] |
| 552 Goettingen [Univ Kl] |
| 663 Valencia [H Univ La Fe] |
| 780 Manchester [Christie] |
| 807 Berlin [Charité Univ] |
| 601 Manchester [Royal Infirmary] |
| 649 Bari [Univ Studi] |
| 740 Linköping [Univ H] |
| 205 London [Hammersmith] |
| 233 Besancon [H Jean Minjoz] |
| 276 Newcastle-Upon-Tyne [Freeman H] |
| 286 Pavia [S Matteo] |
| 574 Olomouc [Univ H] |
| 705 Udine [Univ H] |
| 746 Tartu [Univ H] |
| 161 Tel_Aviv [Sourasky] |
| 763 London [Kings College H] |
| 787 Regensburg [University] |
| 919 Antalya [Medical Park H] |
| 255 Oxford [Radcliffe H] |
| 409 Petach-Tikva [Beilinson H] |
| 183 Tunis [Ctr Nat Gref Moel] |
| 656 Prague [Ist Hematology] |
| 289 Goeteborg [Sahlgrenska Univ H] |
| 644 Vilnius [Santariskiy Kl] |
| 955 Amiens [H Sud] |
| 256 Kiel [UKSH] |
| 369 Beirut [American Univ] |
| 587 Reggio_Calabria [Centro Trapianti] |
| 623 Verona [Policlinico] |
| 677 Katowice [Silesian Med Acad] |
| 825 Alessandria [SS Ant e Bia] |
| 266 Uppsala [Univ H] |
| 580 Amman [King Hussein] |
| 792 Catania [Osp Ferrarotto] |
| 304 Firenze [Careggi-Meyer] |
| 240 Bologna [S Orsola-Malpighi] |
| 386 Bristol [Royal H Sick Chil] |
| 141 Brescia [Civili, Adulti] |
| 208 Zürich [208] |
| 265 Milano [Osp Maggiore] |
| 272 Tours [H Bretonneau] |
| 345 Haifa [Rambam MCH] |
| 768 London [S Bartholomew`s] |
| 238 Córdoba [Reina Sofia] |
| 291 Porto [Inst Oncologia] |
| 303 Cardiff [Univ Wales] |
| 598 San_Sebastian [H Aranzazu] |
| 606 Cuneo [S Croce e Carle] |
| 258 Jerusalem [Univ Hadassah] |
| 268 Belfast [City H] |
| 308 Graz [Medical Univ] |
| 354 Milano [Trap Mid Osseo] |
| 693 Warsaw [Inst Haematology] |
| 823 Plymouth [Derriford H] |
| 254 Leeds [St James] |
| 788 Ancona [Umberto I] |
| 244 Glasgow [Royal Infirmary] |
| 645 Marburg [Philipps Univ] |
| 744 Gent [Univ H] |
| 506 Brugge [AZ Sint-Jan] |
| 617 Ankara [Ibni Sina H] |
| 217 Genova [S Martino] |
| 236 Madrid [Princesa] |
| 294 Milano [Osp Niguarda] |
| 533 Jena [Friedrich-Schiller] |
| 630 Brussels [Univ H] |
| 729 Hradec_Králové [Charles U H, Hem] |
| 307 Rome [Univ S Cuore] |
| 502 Venezia [SS Giovani e Paolo] |
| 584 Barcelona [V d`Hebron Adults] |
| 703 Alger [P et M Curie] |
| 160 Paris [H Necker] |
| 168 Ankara [Hacettepe Univ] |
| 228 Edinburgh [Western General] |
| 302 Zagreb [Univ H Rebro] |
| 642 Oviedo [H Covadonga] |
| 772 Cape_Town [Constantiaberg] |
| 798 Christchurch [Canterbury Health] |
| 262 Paris [Pitie-Salpetriere] |
| 281 Patras [Univ H] |
| 361 La_Coruña [Juan Canalejo] |
| 392 Palermo [Osp V Cervello] |
| 769 Sevilla [Virgen del Rocio] |
| 163 Piacenza [Osp Civile] |
| 290 Karlsruhe [Klinikum] |
| 305 Torino [Regina Margherita] |
| 348 Aachen [RWTH] |
| 501 Liverpool [Royal Univ H] |
| 766 Napoli [Federico II] |
| 786 Mainz [Johannes-Gutenberg] |
| 809 Erlangen [University] |
| 284 Birmingham [Heartlands H] |
| 300 Lisboa [Inst Oncologia] |
| 456 Pretoria [Albert Albert] |
| 589 Adana [Baskent Univ] |
| 610 Bratislava [Univ H] |
| 640 Ljubljana [Univ Med Ctr] |
| 819 Madrid [H G Marañón] |
| 538 Wroclaw [Ctr Cell Transpl] |
| 751 Athens [St. Savas] |
| 282 Valencia [H Clinico] |
| 287 Rome [S C-Forlanini] |
| 526 San_Giovanni_Rotondo [IRCCS] |
| 543 Modena [Policlinico] |
| 646 Roeselare [AZ Delta] |
| 728 Madrid [Puerta de Hierro] |
| 760 Istanbul [Tip Fakueltesi] |
| 868 Lecce [Osp Vita Fazzi] |
| 211 Sao_Paulo [H Sirio-Libanes] |
| 529 Pesaro [O Tranpianti] |
| 558 Munich [Rechts der Isar] |
| 592 Idar-Oberstein [Kl Knochenmarktr] |
| 791 Cagliari [Osp A Businco] |
| 797 Vicenza [Osp S Bartolo] |
| 799 Gdansk [Medical U] |
| 418 Beirut [Makassed Univ H] |
| 539 London [St George`s] |
| 582 Belgrade [Military Med Acad] |
| 628 Izmir [Ege Univ Med Sch] |
| 634 Aarhus [Univ, Hematol] |
| 134 Bonn [Uni] |
| 309 Madrid [Jiménez Díaz] |
| 359 Magdeburg [vGuericke U] |
| 602 Bremen [Kl Bremen-Mitte] |
| 737 Pamplona [Cl Univ Navarra] |
| 816 Warsaw [Military Med Acad] |
| 119 Ascoli_Piceno [Osp Mazzoni] |
| 227 Vienna [Medizinische Univ] |
| 271 Innsbruck [Univ H] |
| 297 Frankfurt am Main [Goethe-Univ] |
| 374 Campinas [Zeferino Vaz] |
| 401 Hangzhou [Univ H] |
| 616 Milano [INT] |
| 622 Athens [Evangelismos H] |
| 691 Ankara [Numune] |
| 699 Wroclaw [Medical Acad] |
| 722 Palma_De_Mallorca [Son Dureta] |
| 931 Suzhou [First Soochow] |
| 299 Bolzano [Osp S Maurizio] |
| 321 Siena [Le Scotte] |
| 338 Halle [Univ Martin-Luther] |
| 412 Ankara [Bayinder H] |
| 660 Reggio_Emilia [S Maria Nuova] |
| 735 Murcia [H M Meseguer] |
| 749 Oldenburg [Klinikum] |
| 862 Ankara [Medicana] |
| 247 Amsterdam [Acad Ziekenhuis] |
| 323 Murcia [V Arrixaca] |
| 332 Taranto [Osp Nord] |
| 530 Greifswald [Ernst-Moritz-Arndt] |
| 784 Essen [Evangelisches Kh] |
| 789 Avellino [S G Moscati] |
| 794 Perugia [Monteluce] |
| 954 Warsaw [Central] |
| 390 Duesseldorf [H Heine U] |
| 615 Madrid [H Ramón y Cajal] |
| 795 Pisa [Az Osp Univ] |
| 970 Flensburg [St Franziskus] |
| 142 Mannheim [Univ] |
| 331 Milano [Inst Eur Onc] |
| 440 Kocaeli [Anadolu] |
| 444 Riyadh [Aziz] |
| 570 Santiago_De_Compostela [H Clin Univ] |
| 607 Napoli [Osp Cardarelli] |
| 652 Tricase_(Lecce) [C Panico] |
| 695 Lublin [Medical Univ] |
| 806 Lyon [H Debrousse] |
| 811 Cagliari [R Binaghi] |
| 858 Jeddah [King Faisal] |
| 930 Moscow [NRC Haem.] |
| 124 Bochum [Knappschafts Kr] |
| 146 Stuttgart [Diakonissen Kh] |
| 153 Hamburg [AK St Georg] |
| 344 Aberdeen [Royal Infirmary] |
| 367 Luebeck [Schleswig-Holstein] |
| 372 Ankara [GATA BMT] |
| 423 Ankara [Oncology R&E H] |
| 427 Bucharest [Fundeni Clin Inst] |
| 428 Gliwice [Sklodowska] |
| 445 Istanbul [Medipol Adult] |
| 512 Cape_Town [UCT Medical Sc] |
| 518 Berlin [HELIOS Kl] |
| 537 Las_Palmas [H Dr. Negrin] |
| 559 Granada [V de las Nieves] |
| 583 Riga [Haematology Ctr] |
| 594 Linz [Elisabethinen H] |
| 627 Kayseri [Erciyes Hem] |
| 710 Perth [Royal H] |
| 712 Wuerzburg [Medizinische Kl II] |
| 761 Istanbul [Cerrahpasa] |
| 861 Potenza [Centro Oncologico] |
| 925 St._Petersburg [FCHBE] |
| 104 Chemnitz [Kl Chemnitz] |
| 187 Aydin [A Menderes U] |
| 190 Frankfurt (Oder) [Clin Internal Med] |
| 197 Bergen [Haukeland Univ H] |
| 245 Parma [Centro Trapianti] |
| 339 Antwerp [AZ Stuivenberg] |
| 402 Gaziantep [Gaziantep U] |
| 434 Singapore [Gen H] |
| 439 Amman [Al Khalidi] |
| 452 Prague [U H Motol] |
| 557 Pavia [S Matteo] |
| 585 Rostock [Kl Inn. Medicine] |
| 590 Berlin [Benjamin Franklin] |
| 591 Minsk [Ctr Paediatric] |
| 612 Cádiz [H del SAS] |
| 621 Izmir [Ege Univ] |
| 668 Barcelona [S Joan de Deu] |
| 686 Eskisehir [Osmangazi] |
| 752 Athens [Agia Sophia] |
| 916 Teheran [Taleghani H] |
| 933 Sheffield [Children H] |
